# The impact of the exposome on cytochrome P450-mediated drug metabolism

**DOI:** 10.3389/fphar.2025.1639646

**Published:** 2025-10-13

**Authors:** Hwaida Ali, Mireille M. Hanna, Noha Alziny, Shuaib Mahmoud, Abdallah Borham, Aya Mustafa, Anwar Abdelnaser

**Affiliations:** ^1^ Biotechnology Graduate Program, School of Sciences and Engineering, The American University in Cairo, New Cairo, Egypt; ^2^ Institute of Global Health and Human Ecology, School of Sciences and Engineering, The American University in Cairo, New Cairo, Egypt

**Keywords:** cytochrome P450 (CYP450), drug metabolism, exposome, environmental exposures, internal exposures, external exposures, drug response variability

## Abstract

The cytochrome P450 (CYP450) family of enzymes plays an important role in drug metabolism, toxicity, and individual responses to medications. Recent research has shown how the exposome, the sum of internal and external exposures during a person’s lifetime, significantly affects CYP450 function. The aim of this review is to investigate how different components of the exposome including external factors such as dietary substances, lifestyle choices, environmental pollutants, and internal factors such as gut microbiota, hormone fluctuations, and disease states, alter CYP450 expression and function, which in turn impacts drug metabolism and treatment outcomes. This review sheds light on the potential of personalized medicine and offers the promise for producing safer and more effective drug therapies in an increasingly dynamic exposure landscape by highlighting the molecular pathways that connect these exposures to alterations in drug metabolizing enzymes and their function.

## 1 Introduction

Drug metabolism is the process by which the body processes pharmaceuticals from initial absorption to distribution to action to their final removal (Ross and Xu, 2021). This process not only has an influence on the therapeutic effectiveness of drugs but also plays an essential role in adverse drug reactions, drug-drug interactions, and interindividual variability in response (Fu et al., 2022).

Drug Metabolism occurs in two phases in the liver: phase I and phase II (Iacopetta et al., 2023). The Cytochrome P450 superfamily plays a vital role in phase I reactions in which the heme-thiolate monooxygenases catalyze the oxidation of lipophilic substrates, facilitating their conversion into more soluble and readily excreted products. CYP450 enzymes metabolize the majority of clinically used drugs, making them a core element in pharmacological and toxicological research (Córdova et al., 2017; Ross and Xu, 2021; Zhao et al., 2021; Hasija, 2023).

The CYP450 family is widely diverse, with multiple isoforms such as CYP3A4, CYP2D6, CYP2C9, and CYP1A2, which promote the biotransformation of a broad range of xenobiotics and endogenous compounds (Dasgupta, 2020). These enzymes are dynamic, which means their expression and activity can be remarkably controlled by both genetic polymorphisms and non-genetic elements, including diet, microbial makeup, stress, and environmental exposures. This diversity contributes to significant interindividual differences in drug metabolism (Hossam Abdelmonem et al., 2024).

Recently, the concept of the exposome has appeared as a potential framework for interpreting how the environment shapes health and diseases throughout life. Initially introduced under the scope of epidemiology of chronic diseases, the exposome includes all non-genetic exposures a human can encounter from the moment of conception (Baluch et al., 2020). This encompasses external exposure such as diet, pollutants, lifestyle habits, drugs, and pathogens, and internal biological factors such as oxidative stress, inflammation, and gut microbiome. The exposome is evolving and cumulative, revealing the complexity of our world where several exposures interact consistently with living systems (Hossam Abdelmonem et al., 2024).

Connecting the exposome to CYP450 activity provides a strong rationale for reevaluating traditional models of drug metabolism. Whereas a lot of focus has been given to genetic influences that affect CYP enzyme’s role, compelling evidence reveals that external environmental exposures are of equal significance in influencing enzyme activity in a biological system. For instance, exposure to tobacco smoke’s polycyclic aromatic hydrocarbons can stimulate the activity of CYP1A1 and CYP1A2, possibly changing the biotransformation of antidepressants and antipsychotics (Zanni et al., 2025). On the contrary, grapefruit juice, a food component, inhibits CYP3A4 and affects the bioavailability of several cardiovascular drugs (Stevens and Kamen, 2019). Moreover, chronic alcohol consumption, stress, inflammation, gut microbiome-derived metabolites can cause either suppression or stimulation of major CYP enzymes, highlighting the highly dynamic nature of drug metabolism (Dempsey and Cui, 2019).

This link between environmental factors and CYP450 activity has direct clinical relevance. Alterations in CYP450 activity can cause toxicity and unexpected side effects, particularly in vulnerable populations. For instance, the influential action of the environment on CYP enzymes can affect the drug development process (Banerjee et al., 2020). Understanding how the exposome shapes CYP450 activity can improve the prediction of drug response across populations and support the design of safer therapies. Personalized medicine seeks to address such variability by tailoring treatments to the individual, but current approaches often emphasize genetic profiling alone. Incorporating exposome-related factors such as diet, pollutants, lifestyle, inflammation, and microbiome status will provide a more comprehensive framework to predict drug response and toxicity. In this context, the exposome enhances the predictive power of CYP450 profiling, enabling more precise drug dosing, reduced adverse effects, and optimized therapeutic efficacy.

Therefore, this review aims to synthesize the current literature on how the exposome interacts with CYP450 enzymes and to highlight its potential role in advancing personalized medicine. By bridging pharmacology and environmental health, this review underscores the advantages of an exposome-informed strategy in both clinical practice and drug development.

## 2 CYP450 enzymes and isoforms

Cytochrome P450 (CYP450) enzymes are key biological catalysts in phase I drug metabolism. They facilitate oxidation of xenobiotics and endogenous compounds via mixed-function oxidase mechanisms, using pyridine nucleotide cofactors as electron donors (Guengerich, 2018). Predominantly located in hepatic microsomes, these enzymes employ activated oxygen to convert lipophilic substrates into more hydrophilic metabolites for elimination or further processing (O’Donnell et al., 2014). Their activity is modulated by both endogenous and exogenous factors, including genetic polymorphisms, sex, age, ethnicity, health status, and exposure to inducers or inhibitors (Waring, 2020).

### 2.1 Major cytochrome P450 family, isoforms and function

The cytochrome P450 enzyme family plays a vital role in the metabolism of various drugs. In humans, this family includes 57 functional genes and 58 pseudogenes. Approximately twelve enzymes primarily from the CYP1, CYP2, and CYP3 families predominantly play a predominant role in metabolizing most xenobiotics, including 70%–80% of all pharmaceuticals currently in clinical use. Highly expressed hepatic isoforms include CYP3A4, CYP2C9, CYP2C8, CYP2E1, and CYP1A2, while CYP2A6, CYP2D6, CYP2B6, CYP2C19, and CYP3A5 show moderate expression, and CYP2J2, CYP1A1, and CYP1B1 are largely extrahepatic. Expression and activity are modulated by genetic polymorphisms, xenobiotics, cytokines, hormones, sex, age, and disease states (Zanger and Schwab, 2013; Zhao et al., 2021). Evolutionary processes such as gene duplication and loss have also shaped CYP gene diversity across species, leading to variations in gene number and subfamily composition (Nelson et al., 2013).

#### 2.1.1 CYP1 family

The human CYP1 gene family comprises CYP1A1, CYP1A2, CYP1B1, and the pseudogene CYP1D1P (Uno et al., 2023). CYP1A1 is expressed in the liver and several extrahepatic tissues including pancreas, thymus, uterus, and the small intestine, CYP1A2 is largely expressed in hepatic, and CYP1B1 is enriched in peripheral tissues such as breast, prostate, and uterus (Dutour and Poirier, 2017). Collectively, CYP1 enzymes metabolize xenobiotics, procarcinogens, eicosanoids, and endogenous compounds including retinol, melatonin, steroid hormones, and 17β-estradiol thus influencing inflammation, detoxification, and carcinogenesis (Divanovic et al., 2013; Mescher and Haarmann-Stemmann, 2018; Stading et al., 2024; Yaqoob et al., 2022; Yuan et al., 2022).

#### 2.1.2 CYP 2 family

The CYP2 family, which includes isoforms such as CYP2A6, CYP2B6, CYP2C8, CYP2C9, CYP2C19, CYP2D6, and CYP2E1, plays a crucial role in the metabolism of drugs and endogenous compounds. Genetic variations within this gene family can substantially influence individual differences in enzyme function and responsiveness to pharmaceutical treatments. The CYP2 family represents the primary epoxygenase isoform that is widely expressed in human endothelial tissue, myocardium, and renal tissue (Shubbar et al., 2024).

CYP2A6 comprises around 4% of hepatic CYP content that has a relatively small active site and metabolizes substances such as nicotine, coumarin, letrozole, tegafur, and several other pharmaceuticals (Tornio and Backman, 2018). CYP2C9 oxidizes xenobiotics and endogenous compounds, with its activity influenced by inducers, inhibitors, and genetic variation; substrates include warfarin, losartan, ibuprofen, and cannabinoids (Daly et al., 2017). CYP2C19, with at least 39 documented alleles, metabolizes benzodiazepines, PPIs, SSRIs, TCAs, voriconazole, and activates clopidogrel, making genotype based dosing clinically valuable (Shubbar et al., 2024). CYP2D6 mediates the biotransformation of around 20% of commonly prescribed drugs, including codeine, tramadol, tamoxifen, metoprolol, and antidepressants (Taylor et al., 2020).

#### 2.1.3 CYP 3 family

The CYP3 family includes the isoforms CYP3A4, CYP3A5, CYP3A7, and CYP3A43 and are primarily responsible for the metabolism of clinical medications. CYP3A4 is the most abundant hepatic enzyme in adults, while CYP3A5 shows variable expression; both exhibit broad substrate specificity and overlapping yet distinct regulatory profiles (Wright et al., 2019). These enzymes catalyze diverse reactions including N-oxidation, C-oxidation, O/N-dealkylation, nitro-reduction, demethylation, dehydration, and C-hydroxylation (Zhang et al., 2024). Their substrates include HMG-CoA reductase inhibitors, anticancer agents, benzodiazepines, immunosuppressants, macrolide antibiotics, anesthetics, and antifungals such as terbinafine (Shang et al., 2024).

While the expression and activity of these CYP isoforms are fundamental to drug metabolism, they exhibit significant interindividual variation, primarily driven by genetic factors.

### 2.2 Genetic variability

Genes encoding CYPs are significantly varied genetically, and various CYP variants are important clinical biomarkers for enhancing drug selection and dosing (Zhou and Lauschke, 2022). Drug metabolism rates differ among individuals due to genetic polymorphisms and differences in the expression of CYP450 enzymes. Xenobiotic metabolism, developmental processes, and treatment responses vary in every individual due to epigenetic modifications, such as DNA methylation, histone modifications, and non-coding RNA regulation, as they play a substantial role in influencing CYP450 enzyme activity (Jin and Zhong, 2023). Based on allele combinations, individuals can be classified as poor, intermediate, extensive, or ultra-rapid metabolizers, which profoundly affects drug efficacy and safety (Riaz et al., 2019). Notably, CYP2D6 shows extensive polymorphism, where 63 alleles are reported, impacting the metabolism of nearly half of its substrate (Kaptsis et al., 2024).

CYP expression is further modulated by ligand-activated receptors such as the aryl hydrocarbon receptor (AhR), pregnane X receptor (PXR), and constitutive androstane receptor (CAR), which control a hepatoprotective gene network in the liver and intestine (Rakateli et al., 2023). Through these receptors, environmental exposures including industrial and agricultural pollutants like phenols, polyphenols, substituted polyphenols, polychlorinated biphenyls (PCBs), polycyclic aromatic hydrocarbons (PAHs), flame retardants, and azoles, induce or suppress CYP enzymes such as CYP1A1, CYP1A2, CYP1B1, CYP2B6, and CYP3A4 (Burkina et al., 2017; Smutny et al., 2013; Wuputra et al., 2025). Hormonal ligands (vitamin D, glucocorticoids, cholesterol derivatives) also regulate CYP activity via PXR, CAR, vitamin D receptor (VDR), and glucocorticoid receptor (GR), influencing both phase I metabolism and transcription of CYP3A5 (Rezen et al., 2011; Noh et al., 2022; Oladimeji et al., 2016).

This receptor-mediated transcriptional regulation represents a primary mechanism by which the exposome dynamically modulates CYP450 enzyme activity, thereby profoundly impacting drug metabolism and response.

## 3 External exposome effects on CYP450 and drug metabolism

Several external environmental factors Including dietary components, environmental pollutants, lifestyle factors, and occupational exposures, create a complex network of interactions that can affect drug efficacy and toxicity (Table 1, Annex 1).

**TABLE 1:** External exposome components and their effects on CYP450-Mediated drug metabolism.

Exposure category	Specific factor	Effect on CYP450/Drug metabolism	References
Dietary and Herbal Modulators	Liver as exposome interface	Liver expresses CYP450s, phase 2 enzymes, and xenobiotic receptors, serving as a primary link between exposome and drug metabolism/detoxification	Barouki et al. (2021)
	Grapefruit juice (Bergamottin)	Inhibits intestinal CYP3A4 → ↑ systemic exposure of substrates (e.g., ↑ felodipine bioavailability)	Dayyih et al. (2024)
	Well-done beef (Heterocyclic amines)	Induces CYP1A2 → ↑ adenoma risk in high NAT2-activity individuals	Voutsinas et al. (2013)
	Turmeric (Curcumin/Piperine)	No clinically significant inhibition of CYP3A, CYP2C9, or acetaminophen conjugation enzymes after short-term use	Volak et al. (2012)
	Nutrient deficiencies (e.g., vitamins)	Alters drug response profiles; an exposome framework enables analysis of nutrient-drug interaction networks	Niedzwiecki et al. (2019)
Environmental Toxicants	Airborne PM_2_._5_ (adsorbed hydrocarbons)	↑ CYP1A1 expression in neutrophils, bronchial epithelium, and alveolar macrophages → oxidative stress, inflammation, apoptosis. Activates AHR → mitochondrial ROS → dysfunction	Li et al. (2023)
	TCDD (Dioxin)	Potently induces CYP1A1 in islets → suppresses glucose-induced insulin secretion, ↑ β-cell death, ↓ plasma insulin	Ibrahim et al. (2019)
Lifestyle Exposures	Tobacco smoke (PAHs)	Dose-dependently induces CYP1A2 (↑2-fold in heavy smokers). Cessation normalizes activity → risk of adverse reactions (e.g., ↓ caffeine clearance). THS/IQOS causes similar ↓	van der Plas et al. (2020)
	Chronic alcohol consumption	↑ CYP2E1 expression → bioactivates toxins (e.g., acetaminophen to hepatotoxic NAPQI). Synergistic induction with fasting	Zakhari et al. (2025), Jiang et al. (2024)
Occupational Hazards	Industrial chemicals (VOCs/solvents)	Modulate CYP expression/function → alter metabolism of drugs/endogenous compounds. Requires comprehensive exposome-based risk assesment	Barouki et al. (2021)

### 3.1 General principle of external exposome

The exposome concept, introduced by Christopher Wild in 2005 as “life-course environmental exposures from the prenatal period onwards” (Miller and Jones, 2014; Wild, 2012). This concept includes all exposures, including chemical, physical, biological, psychological, social, and behavioral factors that shape human health (Barouki et al., 2021). Later, Miller and Jones expanded this framework to include cumulative biological responses to environmental, dietary, behavioral, and internal processes throughout the lifespan. Together, the exposome and the genome help explain variability in both physiological and pathological conditions (Barouki et al., 2021; Miller and Jones, 2014). This concept is particularly relevant to toxicology because it links complex environmental exposures with mechanistic responses. The liver, as the main site of xenobiotic metabolism, expresses a large repertoire of detoxification genes—cytochrome P450s, phase II enzymes, transporters, and xenobiotic receptors—positioning it as a central interface between the exposome and human health (Barouki et al., 2021). The following subsections summarize major categories of external exposome factors and their documented impacts on CYP450 enzymes and drug metabolism.

### 3.2 Examples of external exposomes

#### 3.2.1 External dietary and herbal modulators

Dietary constituents and herbal products can markedly influence CYP450 function. For example, bergamottin in grapefruit juice inhibits intestinal CYP3A4, increasing the systemic exposure of drugs such as felodipine—an interaction with clear clinical implications (Dayyih et al., 2024). Similarly, heterocyclic amines in well-done beef induce CYP1A2, a mechanism implicated in adenoma risk; individuals with high N-acetyltransferase-2 (NAT2) activity and high heterocyclic amine intake show increased risk compared with lower-intake groups (Voutsinas et al., 2013). On the contrary, curcumin from turmeric has been extensively examined for CYP interactions, yet short-term piperine-enhanced curcuminoid formulations showed no clinically significant effects on CYP3A, CYP2C9, or paracetamol conjugation enzymes (Volak et al., 2012). Nutrient status also modifies drug metabolism. Vitamin deficiencies, for instance, can alter metabolic profiles and drug response. Applying the exposome framework allows more comprehensive analysis of such nutrient–drug interactions (Niedzwiecki et al., 2019).

#### 3.2.2 Environmental toxicants

##### 3.2.2.1 Airborne pollutants

There is a complex association between fine particulate matter (PM2.5) exposure and CYP enzyme expression and function as PM 2.5 contributes to an increased CYP 1A1 expression in multiple tissue types including neutrophils, bronchial epithelial cells, and alveolar macrophage, primarily due to adsorbed aromatic hydrocarbons on their surface. Subsequently, this will lead to oxidative stress, inflammatory responses, and cellular apoptosis (Li et al., 2023) Moreover, in recent studies, it was shown that PM 2.5 exposure activates the aryl hydrocarbon receptor, which in turn mediates CYP1A1 overexpression, triggering mitochondrial reactive oxygen species (ROS) generation, thus damaging the mitochondrial membrane and leading to its dysfunction (Li et al., 2023).

##### 3.2.2.2 Persistent organic pollutants

Persistent organic pollutants such as 2,3,7,8-tetrachlorodibenzo-p-dioxin (TCDD) profoundly affect CYP1A1 activity. Both high-dose and repeated low-dose exposures strongly induce CYP1A1 expression in mouse islet cells (Ibrahim et al., 2019). Furthermore, Direct TCDD exposure *in vitro* suppresses glucose-stimulated insulin secretion in human and mouse islets, while high-dose injections lower plasma insulin and increase β-cell death which illustrating how persistent pollutants disrupt metabolic function via CYP-mediated pathways (Ibrahim et al., 2019).

##### 3.2.2.3 Dynamic and regional environmental factors

Beyond persistent environmental factors, dynamic influences such as seasonality and distinctive regional conditions markedly affect CYP450 activity. Seasonal UV exposure has been shown to upregulate CYP1A and CYP1B enzymes through oxidative stress mechanisms, potentially influencing the metabolism of drugs processed by these enzymes. Additionally, living in high-altitude anoxic environments triggers physiological adaptations including hypoxia-induced modulation of CYP450 enzymes, altering drug metabolism rates (Zhou et al., 2018; Hui et al., 2016). These factors may exert cumulative or synergistic effects alongside other exposome factors, thus diversifying individual drug metabolism phenotypes even more, which highlights the importance of their consideration in personalized medicine.

#### 3.2.3 Lifestyle exposures

##### 3.2.3.1 Tobacco smoke

It is known that cigarette smoking significantly induces CYP1A2 activity, with heavy smokers (≥20 cigarettes per day) showing almost a two-fold increase in its activity compared to nonsmokers. This is a result of the exposure to polycyclic aromatic hydrocarbons (PAHs), products of incomplete combustion of organic matter through tobacco smoking (van der Plas et al., 2020). Interestingly, smoking cessation normalizes the CYP1A2 activity, which in turn can lead to adverse drug reactions if not managed appropriately Research demonstrates that after sudden smoking cessation in heavy smokers, caffeine clearance decreased significantly, and this was accompanied by an apparent significant decrease in the half-life of CYP1A2 (van der Plas et al., 2020). Moreover, Alternative tobacco products such as the heat-not-burn tobacco products like the Tobacco Heating System (THS/IQOS) show similar and comparable effects to smoking cessation on CYP1A2 activity. Thus, switching from conventional cigarettes to THS or smoking abstinence results in similar reductions in CYP1A2 activity within 5 days (van der Plas et al., 2020).

##### 3.2.3.2 Alcohol consumption

Alcohol consumption has also been shown to affect the level of CYP enzymes, particularly CYP2E1, as it plays a critical role in ethanol metabolism and toxicant bioactivation. The liver has significant amounts of CYP2E1 expression, which accounts for almost half of all hepatic cytochrome P450 mRNA. Ethanol consumption increases CYP2E1 expression levels (Zakhari et al., 2025). This alcohol-induced upregulation of CYP2E1 has significant implications for drug metabolism. CYP2E1 bioactivates common medications including paracetamol (acetaminophen), producing harmful metabolites like NAPQI that contribute to hepatotoxicity. Fasting and alcohol consumption had a synergistic effect in a study that was done on rats; as rats given both fasting conditions and large amounts of ethanol showed a higher increase in enzyme level compared to rats that were only fasting (Jiang et al., 2024; Zakhari et al., 2025).

##### 3.2.3.3 Occupational hazards

Occupational exposures represent significant contributors to the external exposome affecting drug metabolism. Through this exposome framework we can monitor such occupational risks by conducting comprehensive environmental assessments. Many volatile organic compounds and industrial chemicals that are found in the workplace can modulate CYP enzyme expression and function and thus alter the metabolism of many medications and other endogenous compounds, and the interactions are currently being studied for their effects on the different drug metabolism pathways (Barouki et al., 2021).

**FIGURE 1:**
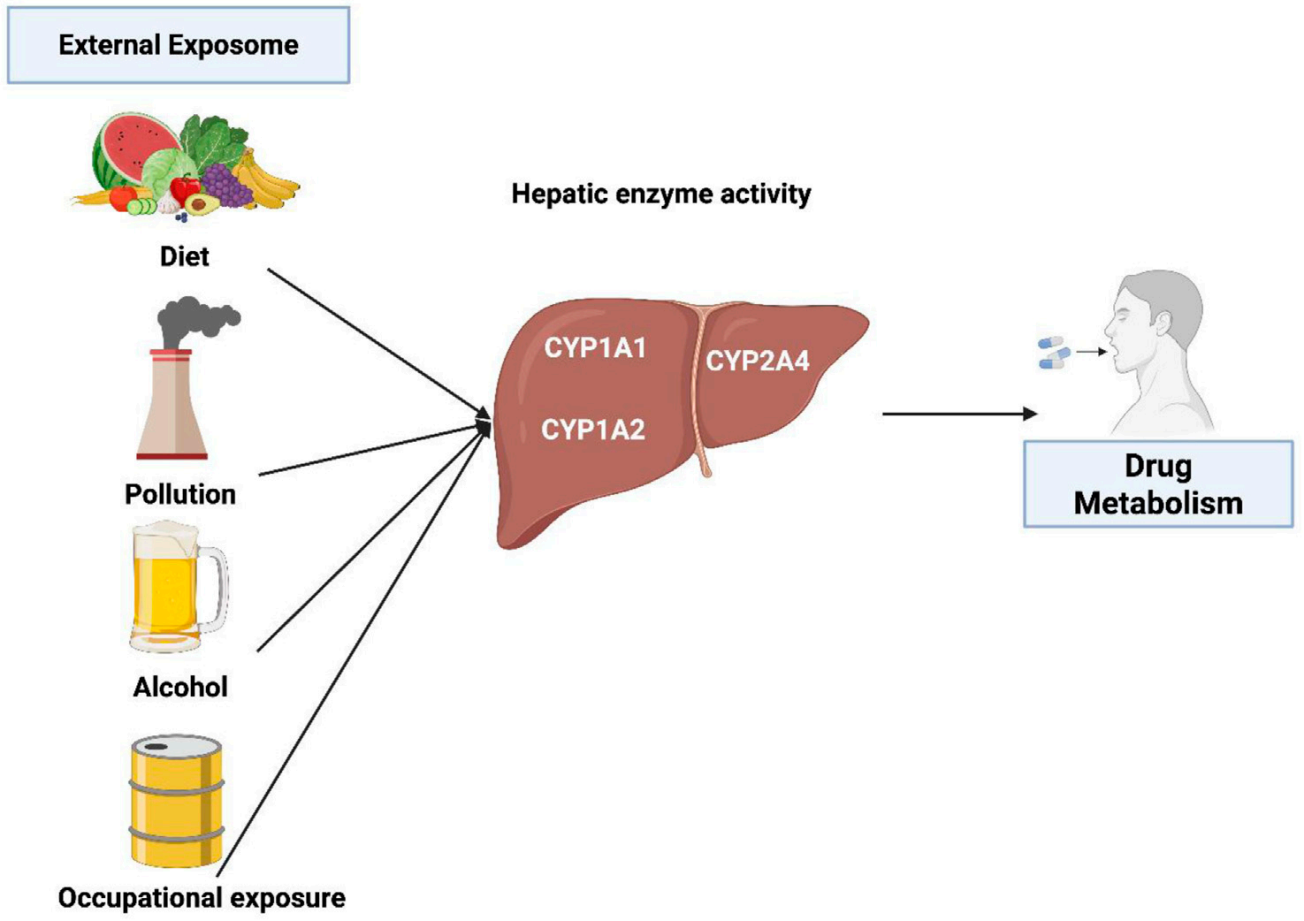
The effect of external exposome on CYP450 enzymes and drug metabolism.

Figure 1 summarizes several key external factors affecting CYP1 activity and drug metabolism.

## 4 Effect the external exposome on the mechanism of drug metabolisms

As previously stated, the exposome includes the totality of environmental exposures that an individual experiences throughout their lifetime, including lifestyle habits, diets, pollution, and more (Buck Louis et al., 2017). These exposures can have a great impact on the drug’s safety and efficacy as well as how the drug is being metabolized (Pristner and Warth, 2020). The drug is mainly metabolized in the liver, where the complex enzymes change the lipophilic substances into more hydrophilic ones that can be easily excreted (Garza et al., 2023). It is essential to comprehend how external factors impact the drug’s metabolism mechanisms to produce a personalized medication (Pristner and Warth, 2020; Yang et al., 2013). There are several ways of how exposomes can affect the mechanisms of drug metabolism either directly or indirectly including enzyme modulation, epigenetics modifications, transporter protein alteration, inflammatory response, and microbiome interactions.

### 4.1 Enzyme modulation

The exposome can lead to induction or inhibition of drug-metabolizing enzymes which in turn affect the drug metabolism. Enzyme induction occurs when the exposome increases the expression of these enzymes, mainly through the activation of nuclear receptors or transcription factors that promote gene transcription (Hakkola et al., 2020). These alterations result in an increase in the capacity of the body to metabolize drugs, which is linked to lower in their plasma levels and, ultimately, a decrease in the therapeutic efficacy of the medication (Pristner and Warth, 2020; Robertson et al., 2012).

**FIGURE 2:**
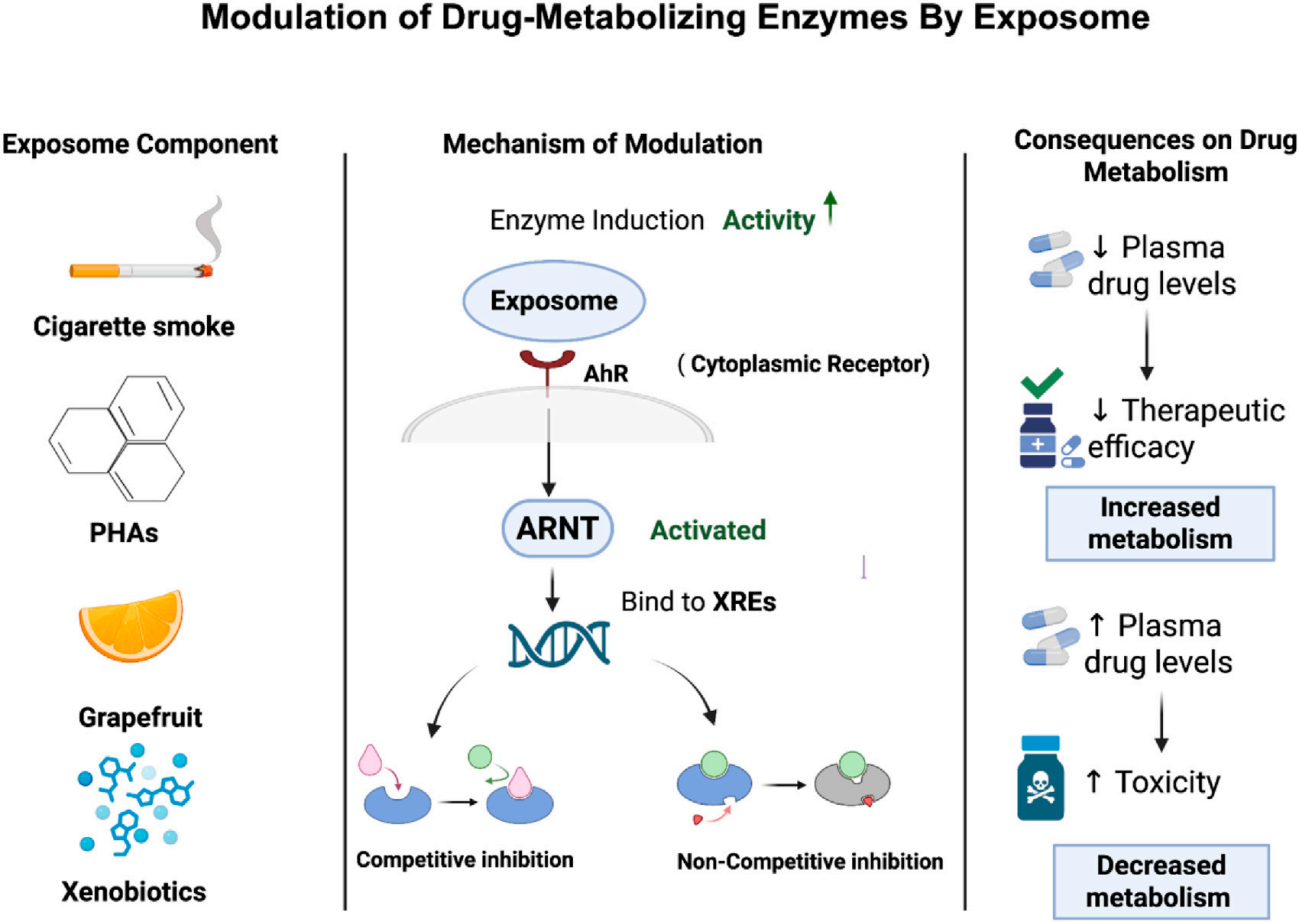
Exposome-mediated modulation of drug-metabolizing enzymes and its impact on drug metabolism. The figure shows how exposome can either accelerate or inhibit drug metabolism, which affects drug efficacy and safety.

For instance, polycyclic aromatic hydrocarbons (PAHs), which are a common exposome seen in cigarette smoke, can bind and activate the aryl hydrocarbon receptor (AhR), which is a cytoplasmic receptor. Upon activation, the receptor moves into the nucleus and forms a heterodimer with the aryl hydrocarbon receptor nuclear translocator (ARNT). This complex can increase the expression of genes that encode for drug-metabolizing enzymes including CYP1 enzymes like CYP1A1 and CYP1B1 in phase I and glutathione S-transferases (GSTs) in phase II through binding to xenobiotic response elements (XREs) within gene promoter regions (Hammond et al., 2022). This leads to increase in the enzymatic activity and accelerates drug metabolism which may lower plasma drug concentrations and reduce therapeutic efficacy especially in drugs that have limited therapeutic windows (Pristner and Warth, 2020). While enzyme inhibition refers to a reduction in enzymatic activity upon exposure to exposomes, which in turn leads to lower drug metabolism. This can happen through direct competitive or non-competitive inhibition or through mechanism-based inactivation (MBI) that is also known as “suicide inhibition”, in which the enzyme is altered irreversibly (Alsanosi et al., 2014). In Figure 2, it summarizes how this mechanistic work ultimately affects drug metabolism. Competitive inhibition happens when exposome components mimic the substrate and compete with the drug for the enzyme’s active site which results in reversible inhibition in which the inhibitor can be replaced by increasing the concentration of drug (Spaggiari et al., 2016). For example, grapefruit medications can act as competitive inhibitors for cytochrome P450 3A4 enzyme (CYP3A4), resulting in reduction of drug metabolism such as simvastatin and increased toxicity in plasma levels (Bailey et al., 2013; Lee et al., 2016). Non-competitive inhibition happens when exposome components bind to an allosteric site which leads to changing in conformation of enzyme and thereby blocking the drug from binding to it which eventually leads to impairment of drug regardless of its concentration (Delaune and Alsayouri, 2022). For instance, it has been demonstrated that flavonoids like 6-hydroxyflavone can act as a non-competitive inhibitor for cytochrome P450 (P450) which affects eventually drug metabolism and leads to drug interactions (Si et al., 2009). Moreover, MBI or suicide inhibition occurs when an enzyme metabolizes a substance and converts it into a reactive intermediate where it forms a covalent bond and binds to the enzyme causing inactivation of it. This leads to permanent loss of enzyme activity until new enzymes are produced (Zhou et al., 2022).

### 4.2 Epigenetic modifications

**FIGURE 3:**
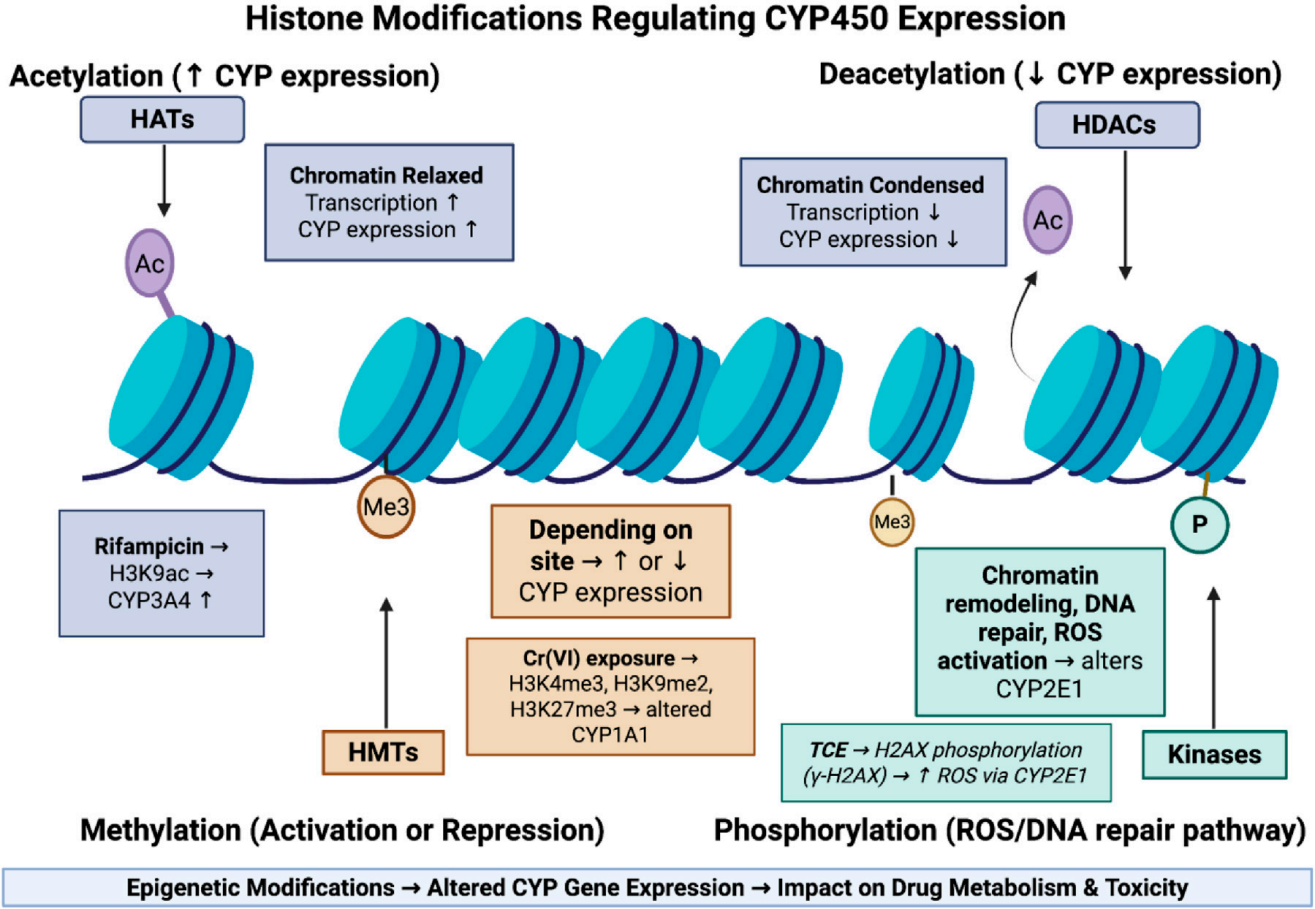
Epigenetic histone modifications regulating CYP450 expression and drug metabolism. This figure shows how histone modifications regulate CYP450 expression by altering chromatin structure. Acetylation relaxes chromatin and increases gene transcription, while deacetylation condenses chromatin and suppresses expression. Methylation can either activate or repress CYP genes depending on the modification site, and phosphorylation is linked to DNA repair and ROS generation, influencing CYP2E1 activity. These epigenetic changes collectively shape how environmental exposures affect drug metabolism.

Epigenetics describes the heritable changes that affect gene expression without altering the underlying DNA sequences. These changes include DNA methylation, Histone modifications, and regulatory non-coding RNAs. The exposomes can induce epigenetic modifications which can alter the expression of genes that are involved in drug-metabolized enzymes, ultimately affecting drug metabolism and its response (Shamsi et al., 2017). This mechanism is summarized in Figure 3.

#### 4.2.1 DNA methylation and drug metabolism

DNA methylation is one of the most important mechanisms that is influencing gene expression that is involves adding a methyl group to cytosine residues specifically at CpG sites that is commonly found in gene promoter sequences, and this addition is maintained by variety of DNA methyltransferases enzymes (DNMTs) (Davletgildeeva and Kuznetsov, 2024). This alteration in methylation pattern can either enhance or inhibit gene expression depending on its location on DNA (Chera et al., 2024). The exposomes can disrupt the normal DNA methylation patterns which leads to hypomethylation or hypermethylation causing an alteration in the expression of genes that encode for drug metabolizing enzymes, thus impairing drug metabolism (Colwell et al., 2023; Rushing et al., 2023). Alteration in methylation patterns in genes that encode for CYP can lead to a decrease in drug metabolism or shifting activity of enzymes (Colwell et al., 2023). For instance, a study by Liu et al. (2022b), has demonstrated that mycotoxin deoxynivalenol (DON) that is commonly found in contaminated food can alter DNA methylation pattern of genes that is involved in drug metabolism by increasing expression of several CYP450 enzymes like CYP1A1, CYP2E1, and CYP3A29 which eventually disrupting normal liver function and drug metabolism. Moreover, a study by Liu et al. (2022a) has shown that exposome including Benzoapyrene (BaP) can alter methylation patterns and affect drug-metabolizing enzymes in human bronchial epithelial cells. The results have revealed that BaP exposure reduced DNA methylation pattern in the promoter region of the CYP1A1 gene which accelerate the metabolism of toxins, but it has been noted that GSTP1 showed increased methylation which reduce its expression that is in turn lead to suppression of detoxification ability (Liu et al., 2022a). These findings shed light on how exposome could affect DNA methylation of drug-metabolizing enzymes and cause impairment in drug metabolism and detoxification.

#### 4.2.2 Histone modifications and drug metabolism

Histone modifications refer to the chemical changes that occur on histone proteins, in which DNA is tightly wrapped around them. These changes have a significant impact on chromatin structure and regulation by altering DNA’s accessibility to transcription factors, which in turn control gene expression. These changes include phosphorylation, methylation, and acetylation (Klibaner-Schiff et al., 2024). The exposome can change histone modifications which impact expressions of genes encode the enzymes that are involved in drug metabolism (Tomkiewicz et al., 2024).

Histone acetylation refers to the addition of acetyl groups to the lysin residue on the histone tail by an enzyme known as Histone acetyltransferases (HATs), in which it neutralizes the positive charge of lysin, relaxes the chromatin structure, and promotes gene transcription (Peng and Zhong, 2015). Deacetylation involves removing acetyl groups using Histone deacetylases (HDACs), which make chromatin more condensed and cause gene suppression (Peng and Zhong, 2015). The exposome can induce acetylation or deacetylation on the promoter region leading to alteration in drug-metabolizing enzymes. For example, a study by Yan et al. (2017) has that exposome component of rifampicin can the expression of CYP3A4 through increasing the acetylation of histone H3 at lysine 9 (H3K9ac) at promoter region which in turn leads to loosen chromatin structure and promote gene transcription.

Histone methylation involves the addition of methyl groups to lysine or arginine residues on histone tails which is regulated by histone methyltransferases (HMTs). Depending on location, it can cause activation or repression of gene expression (Peng and Zhong, 2015). The exposure to an exposome can alter the pattern of this modification. For example, chronic hexavalent chromium [Cr (VI)] exposure has been shown to elevate histone methyltransferases, thereby increasing H3K4me3, H3K9me2, and H3K27me3 marks (Wang et al., 2018). These epigenetic alterations may impair transcription factor recruitment and alter expression of drug-metabolizing genes such as CYP1A1, ultimately impacting drug metabolism and pharmacokinetics (Wang et al., 2018).

Histone phosphorylation is an epigenetic mechanism that uses kinases to add phosphate groups to serine, threonine, or tyrosine residues on histone proteins. This modification is mainly linked to chromatin remodeling that occurs during transcriptional activation, DNA repair, and cell cycle progression. Exposure to the exposome can alter this process in which it affects drug metabolism (Klibaner-Schiff et al., 2024). For example, A study by Toyooka et al. (2018), demonstrated how exposure to trichloroethylene (TCE) leads to the phosphorylation of histone H2AX at CYP2E1 gene which affects drug metabolism. The results have revealed that TCE exposure alters phosphorylation of H2AX (γ-H2AX) which in turn increases ROS production through CYP2E1 and leads to Activation of DNA damage response.

**FIGURE 4:**
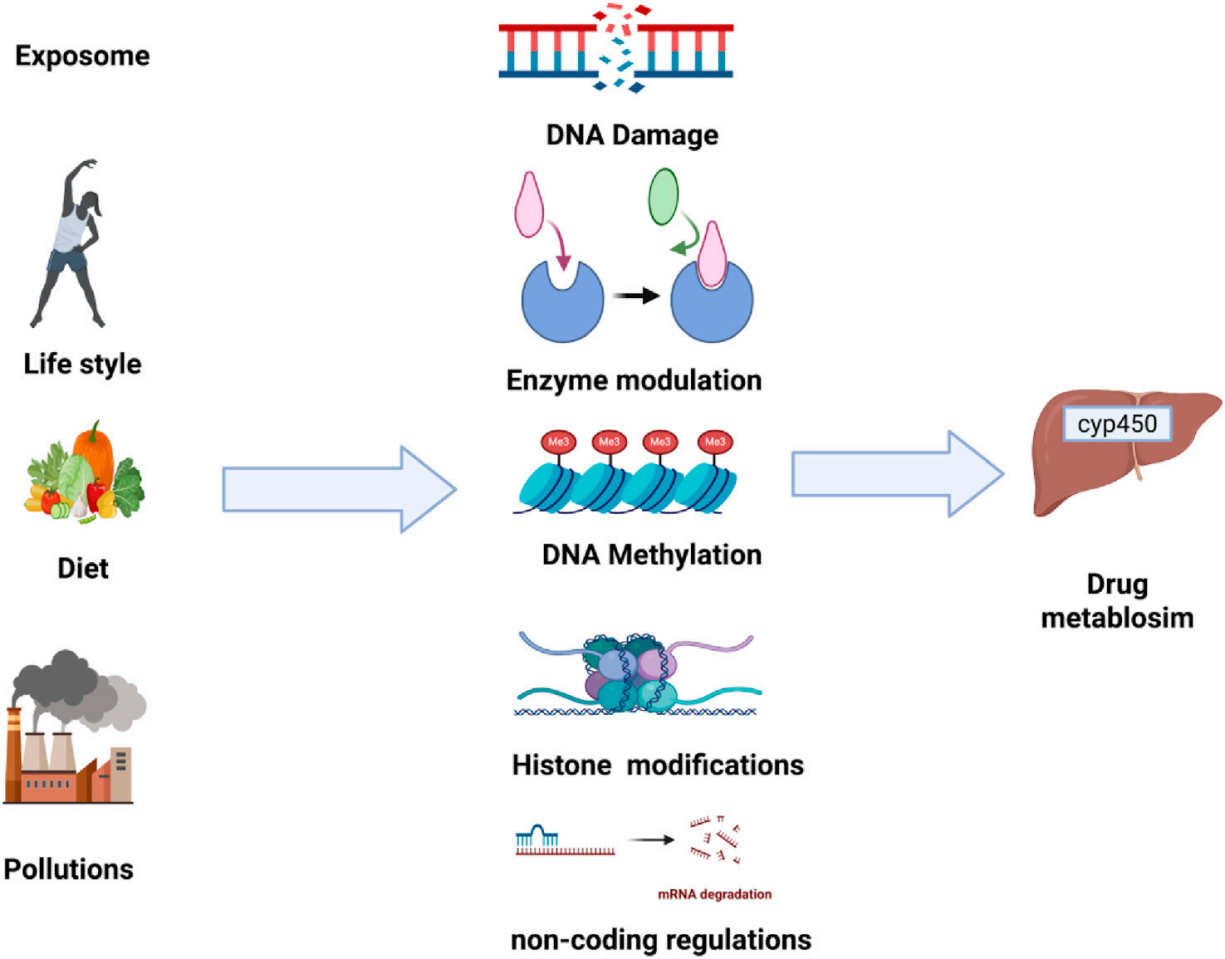
The effect of external exposome on drug metabolism mechanism.

Collectively, these findings highlighted how the exposome can trigger epigenetic changes including acetylation, methylation, and phosphorylation which affect drug metabolizing enzymes that in turn influence the drug metabolism and toxicity which is summarized in Figure 4.

### 4.3 Non-coding RNAs and drug metabolism

Non-Coding RNAs are RNA molecules that do not code for proteins, but they are involved in gene regulation at transcriptional and post-transcriptional levels. Among them, microRNAs (miRNAs), and long non-coding RNAs (lncRNAs), in which they play an important role in modulation of the expression of drug-metabolizing enzymes such as CYP450. MiRNAs are short nucleotides RNAs in which they bind to the 3′untranslated regions (3′UTRs) of target mRNAs, leading to their degradation or translational repression. While lncRNAs can regulate gene expression through various mechanisms, such as chromatin remodeling and transcriptional interference. The exposome can significantly alter the expression and activity of those non-coding RNAs. For instance, a study by Liu et al. (2020), has shown that the exposure of BPA can alter miRNA, specifically miR-27b-3p which in turn has an effect on drug metabolism. The results have shown that BPA causes a downregulation of miR-27b-3p which leads to an increase in the expression of CYP1B1 and subsequently promotes oxidative stress and apoptosis in spleen lymphocytes. Similarly, a study by Chuturgoon et al. (2014) investigates how exposure of Fumonisin B1 (FB1) alters drug metabolism through the regulation of miRNA in human liver cells.

Researchers have demonstrated that FB1 exposure leads to the downregulation of miR-27b that leads to an increase in CYP1B1 expression. This upregulation of CYP1B1 has an influence on drug metabolism and increases production of ROS, which contributes to toxicity. Moreover, lncRNAs have been involved in regulating gene expression of drug metabolizing enzymes. However, studies on how lncRNAs affect drug metabolism are very limited. One of the studies shows how benzo[a]pyrene (B[a]P) influences lncRNAs through activation of the aryl hydrocarbon receptor (AhR) in human lung epithelial cells. The results have shown that exposure to B[a]P causes an activation of AhR receptor through different lncRNAs including SATB1-AS1, MIR4290HG, AC008969.1, LINC01533, and VIPR1-AS1. Some of these lncRNAs are known for their ability to regulate CYP1A1 and CYP1B1. These findings suggest that exposure to environmental toxins like B[a]P can induce AhR activation through alteration of lncRNAs expression, which in turn has an influence on drug-metabolizing enzymes and affect drug responsiveness and toxicity.

Current research on external exposome effects on CYP450-mediated drug metabolism reveals critical knowledge gaps. First, while individual exposures (e.g., air pollutants, dietary phytochemicals) are well-characterized, synergistic effects of mixed exposures remain understudies (Barouki et al., 2021; Tomkiewicz et al., 2024). Secondly, non-chemical stressors (e.g., socioeconomic factors, circadian disruption) are rarely integrated as factors in the research studies even though they modulate CYP activity through inflammatory pathways like IL-6-mediated CYP1A2 suppression (Wang et al., 2022). Additionally, more studies should focus on population-specific vulnerabilities as they are inadequately described, particularly in groups with combined genetic polymorphisms (e.g., CYP2C19 poor metabolizers) and high occupational toxicant exposure (Chen et al., 2012). Finally, the mechanistic links between environmental exposures and epigenetic CYP regulation (e.g., DNA methylation by heavy metals) require deeper exploration and understanding so we would be able to predict individual metabolic variability (Chen et al., 2012).

## 5 Internal exposome effects on CYP450 and drug metabolism

Not only external factors can affect drug metabolism but also Several Internal exposomes can affect drug responsiveness including gut microbiome, inflammation, endogenous hormones, disease states, and internal omics, (Table 2, Annex 1) (Janssen et al., 2025).

**TABLE 2:** Internal exposome components and their effects on CYP450 -mediated drug metabolism.

Exposure category	Specific factor	Effect on CYP450/Drug metabolism	References
Gut Microbiome	Desulfovibrionales bacteria	Produces H_2_S → activates FXR → inhibits CYP7A1 (atorvastatin metabolism)	Hu et al. (2022), Fu et al. (2014)
	Akkermansia muciniphila disruption	Statins alter microbiome composition; linked to altered drug response	Cheng et al. (2022)
	Segmented filamentous bacteria (SFB) and IL-22	Regulates CYP3A11 expression in mice (75% of murine drug metabolism)	Degraeve et al. (2023)
	Antibiotic-induced dysbiosis	↓ CYP3A11 activity; shifts in microbial taxa (Akkermansia, Alistipes) correlate with tacrolimus AUC	Degraeve et al. (2023)
Inflammation and Oxidative Stress	IL-6	Downregulates CYP3A4 (most sensitive), CYP2C9, CYP2C19 via MAPK/ERK and PI3K/AKT pathways	Reeh et al. (2019), Keller et al. (2016)
	IL-1β	↓ CYP3A4 mRNA by 95% via NF-κB pathway; minimal effect on CYP2C9/19	Zhou et al. (2016)
	TNF-α	↓ CYP3A4 mRNA via NF-κB and MAPK/ERK; no protein-level effect	Shi and Sun (2018)
Hormones	Pregnancy	↑ CYP3A4 (nifedipine clearance), ↑ CYP2D6; ↓ CYP1A2 and CYP2C19 (↑ toxicity risk for psychotics/asthmatics)	Betcher and George (2020)
	Quinestrol (exogenous estrogen)	↑ CYP4A1 transcription via nuclear receptor binding; alters mRNA stability	Su et al. (2017)
	Quinestrol + Clarithromycin	↓ Hepatic CYP450 content vs. quinestrol alone (CYP3A4 inhibition)	Ji et al. (2024)
Disease States	Liver cirrhosis	Architectural disruption → ↓ CYP450 activity (reduced hepatocytes/blood flow)	Hirano et al. (2015)
	Type 2 Diabetes (T2D)	↓ CYP3A4 (1.6-fold), ↓ CYP2C19, ↓ CYP2B6 activity; ↓ midazolam/testosterone metabolism	Gravel et al. (2019)
	HIV/Crohn’s/hepatitis C/cancer/RA	↓ CYP3A activity	Coutant and Hall (2018)
	Glioma (brain tumor)	↑ CYP1A1 expression → DNA disruptors → carcinogenesis	Wang and Zuo (2018)
	Brain CYP distribution	Isoforms (CYP1/2/3/46A1) localized in basal ganglia, amygdala, etc.; pathology alters expression	Sjöstedt et al. (2020), Durairaj and Liu (2025)

Internal exposomes interact with the genome through epigenetic modification, affecting drug metabolism without affecting the genome itself. In recent research performed by Messner et al. (2018) on three-dimensional (3D) liver microtissue prepared from primary human hepatocytes (PHH) in coculture with nonparenchymal cells (NPCs) to assess cytochrome activity for one incubation time (24 h) with CYP-substrates. They tested the inducibility with phenobarbital, omeprazole, and rifampicin for 24 h. However, it was found that phenobarbital induced CYP3A4, CYP2B6, and CYP2C19. Omeprazole was specific to CYP1A2, Rifampicin-induced CYP3A4, 2C9, to a lesser extent to CYP2C19. Implicitly, CYP450 activity is highly impacted by the CYP-substrates that are considered internal exposomes because this *in vitro* system acquires many *in vivo* properties. Thus, cells are oriented in a 3D complex network, and they can interact with different cell types, which gives careful evaluation of system interactions (Messner et al., 2018). Hence, we can consider those inducers as internal exposomes.

### 5.1 Gut microbiome

One of the internal exposome that affect drug metabolism is the gut microbiome. The gut microbiome is a highly complex and diverse biological ecosystem, comprising 5 million genes and 100 trillion cells. While it includes multiple dominant phyla such as Verrucomicrobia, Fusobacteria, Proteobacteria, Bacteroidetes, Actinobacteria, and Fusobacteria, the two phyla Firmicutes and Bacteroidetes dominate its composition (Dikeocha et al., 2022). It is known that the Microbiome can only be affected by xenobiotics; however, we aim to explore the complete opposite and the culmination of both counterparts affecting each other endlessly, paving the way to the internal exposome, wide effects, and interactions with drug molecules inside the human body. That said, and as the gut microbiome influences drug metabolizing enzymes, particularly CYP450, we recognize it as an “internal exposome”.

For instance, in a recent research study the authors collected fecal samples from cholesterol gallstone patients and conducted gut microbiome analyses in which they found a higher abundance of Desulfovibrionales bacteria. Moreover, subsequent implantation of Desulfovibrionales in mice was performed, and the gene expression of cholesterol 7α-hydroxylase (CYP7A1) was measured. The inhibition of CYP7A1 was attributed to Desulfovibrionales metabolites. Desulfovibrionales have genes for sulfate reduction that reduce sulfate to hydrogen sulfide, which results in enhancing farnesoid X receptor (FXR) in HepG2 cells and followed by CYP7A1 inhibition (Hu et al., 2022). Importantly, CYP7A1 is the metabolizing enzyme of atorvastatin, which is an effective drug for cholesterol lowering in cardiovascular disease (Fu et al., 2014). However, it has been found that statins themselves alter gut microbiomes, as evidenced by Akkermansia muciniphila disruption in mice (Cheng et al., 2022).

Further evidence linking drug metabolism and gut microbiome is research investigating how tacrolimus (TAC) pharmacokinetics were affected by gut microbiome using *in vivo* mice models. Mainly, CYP3A11 and CYP3A13 metabolize TAC, along with an ATP-dependent drug efflux pump (ABCB1) that facilitates its excretion from the gut to the liver for further metabolism. Here, the ABCB1 gene was affected by gut microbiota in mice. In this study, it was reported that the gut microbiome in the intestines reduced the activity of CYP3A11 in the antibiotic mice group, whereas CYP3A13 remained unaffected. Strikingly, ABCB1 expression in distal, median, and proximal segments of the small intestine was significantly correlated with TAC area-under-the-curve (AUC) values, paralleling shifts in microbial taxa (e.g., high abundance of Akkermansia and Alistipes and low abundance of Oscillibacter and Ruminococcus) under the administration of TAC (Degraeve et al., 2023). Given that CYP3A11 is responsible for 75% of drug metabolism in mice, regulation is critical. Follow-up studies revealed that the regulation of CYP3A11 is modulated by both the production of interleukin-22 produced by innate lymphocyte cell 3 (ILC3) in small intestine epithelium, and the presence of the segmented filamentous bacteria (SFB) which collectively affect the expression of CYP3A11, highlighting the mechanistic link between microbial communities and drug metabolism through *in vivo* models. Cardiac research reported that aspirin alters the gut microbiome and is responsible for the impact of Trimethylamine N-oxide (TMAO), a byproduct of carnitine and betaine, on platelets (Brown and Pereira, 2018). Therefore, more focus should be made on microbiota-immunity crosstalk and its effect on drug metabolism and toxicity (Flannigan et al., 2020).

### 5.2 Inflammation

Inflammation markedly affects drug metabolism by downregulating CYP450 enzymes, a process often termed phenoconversion. Its extent depends on factors such as the type and severity of inflammation, genetic variability, and drug metabolic pathways (de Jong et al., 2020). Among inflammatory markers, Interleukin-6 (IL-6) is the most extensively studied, showing strong suppression of CYP2C9, CYP2C19, and especially CYP3A4 in primary human hepatocytes (PHHS). IL-6 acts primarily through three pathways: JAK/STAT3, MAPK/ERK, and PI3K/AKT, with MAPK/ERK and PI3K/AKT being the most significant regulators of CYP downregulation (Reeh et al., 2019; Keller et al., 2016).

Other cytokines also modulate CYP expression. Interleukin-1β (IL-1β) reduces CYP3A4 mRNA levels by ∼95% via NF-κB activation but has minimal effects on CYP2C9 and CYP2C19 (Zhou et al., 2016). Tumor Necrosis Factor α (TNF-α) downregulates CYP3A4 mRNA selectively through NF-κB and MAPK/ERK pathways, although without altering protein levels (Shi and Sun, 2018).

### 5.3 Endogenous hormones

The endocrine system secretes hormones to coordinate and regulate the human body’s internal metabolism, including drug metabolism, growth, development, and response to injury, stress, or environmental stressors. The glands (e.g., thyroid, parathyroid, pituitary, adrenal) secrete many hormones. Additionally, the female hormones (e.g., estrogen and progesterone) are mainly secreted from the ovaries.

Importantly, pregnancy-induced hormonal fluctuations significantly modulate CYP450 activity. Notably, CYP3A4, responsible for metabolizing 50% of clinically used drugs, shows elevated activity during the pregnancy period, as observed in the enhanced clearance of antihypertensive drug nifedipine. Similarly, CYP2D6 activity rises during the second and third trimesters, whereas CYP1A2 and CYP2C19 activity decline during pregnancy. This suppression of CYP1A2 is relevant, as it metabolizes asthmatic and psychotic drugs, potentially increasing the risk of drug toxicity (Betcher and George, 2020). These adaptations are attributed to the pregnancy-associated hormones, though the underlying mechanisms by which endogenous sex hormones regulate CYP activity remain poorly understood.

For instance, Mongolian gerbil studies have reported that quinestrol, an exogenous estrogen, modifies metabolizing enzymes. Quinestrol binds to nuclear receptors, which then interact with specific response elements in the promoter region of CYP genes. Specifically, CYP4A1 transcription was enhanced, leading to alterations in drug metabolism. It also influences the stability of mRNA transcripts. Notably, progesterone influenced CYP activity, whereas testosterone had little effect. Further studies are required to investigate the underlying mechanisms (Su et al., 2017).

Further evidence investigating the impact of quinestrol on male mice stated that CYP450 content per gram of liver was higher than the control group. While the combined treatment (quinestrol + Clarithromycin) resulted in lower CYP450 content than the quinestrol group. This could be justified by the fact that Clarithromycin’s inhibition of CYP3A4 might lower the turnover rate of the enzyme in the liver, leading to reduced CYP450 content per liver gram (Ji et al., 2024).

### 5.4 Disease states

Disease states significantly influence CYP450-mediated drug metabolism. Liver diseases, particularly cirrhosis, suppress CYP activity due to parenchymal changes, reduced blood flow, and loss of functional hepatocytes, leading to impaired drug metabolism and detoxification (Hirano et al., 2015).

Type 2 Diabetes (T2D) alters CYP activities in an isoform-dependent manner: CYP2C19, CYP2B6, and CYP3A4 activities are reduced by 1.5–2-fold, whereas other isoforms may be unaffected or slightly increased (Gravel et al., 2019). Given that CYP3A4 metabolizes nearly 40% of therapeutic agents, reduced activity in T2D patients, shown by a 1.6-fold decrease in hepatic CYP3A4 levels and substrate metabolism, underscores the importance of caution with polypharmacy, as disease–drug–drug interactions may occur (Coutant and Hall, 2018).

The brain also expresses resident CYP enzymes at the tissue level, the blood-brain barrier, and the cerebrospinal fluid barrier, with roles in xenobiotic metabolism, homeostasis, and disease modulation (Fanni et al., 2021). In glioma, for example, CYP1A1 is upregulated, generating reactive intermediates that may contribute to carcinogenesis (Wang and Zuo, 2018). Brain CYP distribution is extensive—across the cortex, basal ganglia, hippocampus, substantia nigra, and other regions—with several isoforms (CYP1, CYP2, CYP3, and the brain-specific CYP46A1) expressed in astrocytes, neurons, and endothelial cells (Sjöstedt et al., 2020). Consequently, neuropathologies may alter CYP expression locally, further linking disease states and CYP-mediated metabolism (Durairaj and Liu, 2025).

### 5.5 Combined exposures: internal factors can interact with each other

The interconnectedness in the internal exposomic factors is not yet posited; however, one can postulate the intricate connections between bodily response, and reactions, accumulating into grandiose, singular severe effect on the CYP450 enzymes all over the body, augmenting endogenous and exogenous substance metabolism, especially drug metabolism and breakdown. Among many systems inside our body, two were previously touched deeply, CNS and Liver, as such we came across multiple other factors in other systems and intersystem drivers for a polyexposomic interference with drug metabolism by CYP450 interactions. CYP-led reactions in the CNS can conclude an orchestra of different ROS products which disrupt by default the neuroprotective mesh of the brain and the CNS, leading to initial neurotoxicity that eventually led to neurodegeneration and disease progression (Miller et al., 2017).

Inflammation and ROS production from varying reasons such as oxidative stress and damage, activation of microglia and astrocytes, chemokine-mediated and cytokine-mediated inflammatory reactions, all can affect the expression of CYP450, consequently, mediating drug efficacy and toxicity mechanisms (Mast et al., 2021). Liver functions vary which explains its close proximity to being one of most important organs that hosts most of the phase 2 enzymes involved in drug metabolism. Intriguingly, the “pancreas-liver” axis answers many metabolic questions as well as many GITS chronic diseases with the full assistance of “gut-liver” axis, and finally the gut microbiota, this is the full orchestral crosstalk that initiate many consequences on the metabolism of different substances. A possible mechanism is lipid homeostasis disruption, with AhR, PXR, and CAR, all of these actors of the nuclear metabolic receptor superfamily are involved in developing liver steatosis that has further serious implications, leading to structural and biochemical damage of the liver parenchyma; hence, dysregulated drug metabolism as a result of dysfunctional CYP450 enzymes (Bourganou et al., 2025).

An altered microbiome will cause a dysfunctional gut barrier which will affect the first responder to xenobiotics; the liver, causing a metabolic disruption in the liver. The increased permeation of the gut barrier will allow substances such as bioactive lipids, and short-chain fatty acids, to induce inflammatory reactions in the liver exemplified in IL-1β, IL-6, and TNF-α (Barouki et al., 2021). Gao et al. investigated the core components of both internal and external exposomes, in what they called inter-omics analysis between episome, microbiome, proteome, and metabolome, as a result, they deciphered some of the mysteries underlying external-internal exposome interactions (Gao et al., 2022).

They postulated that air quality index correlates with personal creatinine, aminotransferase, cysteinyl proline, monoglyceride, adiponectin, and immunoglobulin lambda. Unexpectedly, fungi came out as the most prominent biological component in the external exposome-proteome and external exposome-metabolome interactions, but no clear human health clues. A major outcome of these analyses is finding that multiple systems such as the immune system, kidney, and the liver play significant roles in the exposomic interactions, as they’re well-known in dealing with both phases; internal and external exposures. Complement C3, IL-1 receptor, and Immunoglobulin proteins were the most highly annotated components from the external exposome-proteome analysis. L-arginine, uracil, and other amino acids were the highest degree metabolites detected in the external exposome-metabolome array. Alanine aspartate, glutamate metabolism, protein absorption and digestion, and beta-alanine metabolism are the highest degree immune-related pathways which were also involved in external exposome-metabolome analysis (Gao et al., 2022).

Moreover, we previously highlighted that Eggerthella members were likely contributing to diseases like liver disease, ulcerative colitis, and systemic bacteremia. Parasutterella was also involved in bile acid metabolism and regulation. Roseburia, Alistipes, Eggerthella, Odoribacter, Parasutterella were all involved in proinflammatory activities that promote chemical stressors, leading to systemic inflammation and increased susceptibility to disrupted systems, causing various diseases, and affecting drug metabolism (Parker et al., 2020).

**FIGURE 5:**
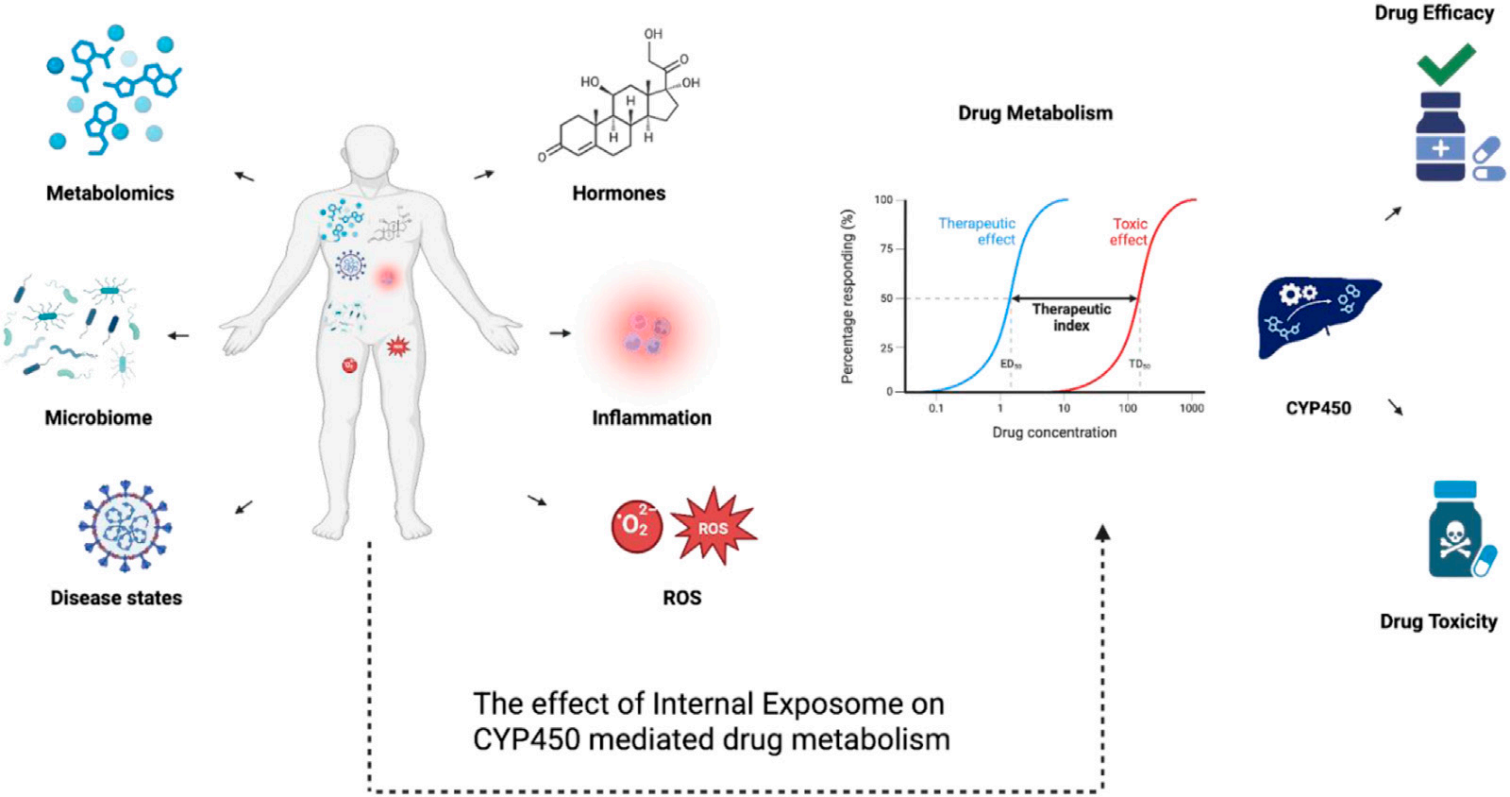
A diagram showcasing the multifactorial effects of internal exposome on CYP450-mediated drug metabolism. Internal exposome factors including disease states, inflammation, reactive oxygen species (ROS), hormones, the microbiome, and metabolomic alterations can regulate CYP450 expression and its activity through transcriptional, post-transcriptional, and post-translational mechanisms. These regulatory mechanisms can affect CYP450-mediated drug metabolism, shifting therapeutic index and impacting drug efficacy or toxicity.

In essence, the various multifactorial interactions between different Internal Omes showcases the complexity of this myriads of components that autonomously interact with each other causing disruption in biological processes, full spectrum inflammation, and consequently, dysregulated drug metabolism (Parker et al., 2020). To correlate a plethora of chemical and biological components under the umbrella of unlocking internal and external exposome, Figure 5 explains how factors such as the gut microbiome, disease states, hormonal fluctuations, inflammation, and oxidative stress (ROS) affect phenotypes and biochemical pathways in different systems inside the body. Additionally, these factors shift the therapeutic index and affect both treatment efficacy and cytotoxicity through influencing cytochrome P450 activity, which converges on drug metabolism. That mentioned, isolating these proceedings and associating them with drug metabolism, more importantly, health outcomes is the main challenge.

## 6 Implications for drug toxicity and personalized medicine

 The exposome-encompassing environmental exposures, lifestyle factors, and genetic interactions-profoundly influences CYP450 enzyme activity, creating variability in drug response and toxicity, which necessitates advanced strategies for personalized medicine that integrates genetic, biomarker, and environmental data to optimize therapeutic outcomes.

Personalized medicine seeks to tailor treatments based on individual differences to improve drug efficacy and safety. A major challenge addressed by personalized medicine is variability in CYP450-mediated drug metabolism, influenced by genetic polymorphisms. However, beyond genetics, environmental exposures—diet, pollutants, lifestyle factors, collectively termed the exposome—significantly modulate CYP450 activity and thus drug response. Integrating exposomic data with pharmacogenomics offers a more complete understanding of the factors shaping drug metabolism phenotypes, improving therapeutic precision (Alshamrani et al., 2024).

The exposome accounts for dynamic environmental influences that alter CYP450 enzyme function, which genetic data alone cannot capture. This leads to better predictions of drug metabolism variability, optimization of drug dosing, and reduction of adverse drug reactions. Hence, exposomic insights complement genomic data in delivering truly personalized treatments tailored to each patient’s unique genetic and environmental context (Alshamrani et al., 2024).

### 6.1 Genetic-exposome interplay

Environmental factors can interact with the genetic polymorphisms in CYP450 enzymes causing alteration in drug metabolism. For example, CYP2C19 ultra-rapid metabolizers when exposed to smoking exhibit a much greater clopidogrel activation, increasing bleeding risk due to enhanced prodrug conversion (Cheng et al., 2023). Similarly, for CYP1A2 slow metabolizers when they consume charred meat face an almost 5 times increase in esophageal cancer risk, as PAHs in charred foods are inadequately detoxified in their bodies (Gastelum et al., 2020; Wang et al., 2012). Also, gene-environmental epistasis further modulates detoxification pathways. GSTP1 variants reduce glutathione conjugation efficiency, impairing CYP1A1-mediated PAH detoxification and thus, increasing cancer susceptibility in smokers (Xu et al., 2013). Concurrently, UGT1A128 polymorphisms exacerbate irinotecan toxicity in urban populations exposed to PM, which induces CYP3A4-mediated bioactivation of the drug (Wang et al., 2022; Xu et al., 2013).

### 6.2 Exposure biomarker integration

Advanced phenotyping methods, such as S-mephenytoin/CYP2C19 activity ratios can reveal enzyme inhibition in pesticide-exposed agricultural workers, which can be used to guide dose adjustments in those individuals (Wang et al., 2022). Omics-based approaches, such as metabolomic profiling of PAH-CYP1A1 DNA adducts, provide quantitative exposure biomarkers that can link environmental PAH levels to metabolic dysfunction (Samer et al., 2020).

Dynamic Monitoring Systems Wearable sensors that track real-time ozone exposure in correlation with CYP2E1 induction, facilitates dynamic dosing adjustments for drugs metabolized by this enzyme (Wang et al., 2022). Geo-tagged electronic health records can map urban air pollution and identify exposed populations who would have suppressed CYP1A2 activity, thus requiring therapeutic modifications and dose adjustments (Samer et al., 2020).

### 6.3 Clinical implementation strategies

Recent advances in precision environmental health monitoring have demonstrated the power of integrating longitudinal exposome profiling with multi-omics analyses to capture the individualized nature of environmental exposures and their biological consequences (Gao et al., 2022). Researchers measure thousands of chemical and biological components in a single individual’s personal exposome and correlate these with internal biomolecular changes, including proteomics, metabolomics, and clinical markers. This revealed that even individuals living in the same geographic area can have highly distinct and fluctuating exposure profiles, with agrochemicals and fungi frequently dominating the external exposome. They also found strong correlations between specific environmental exposures and changes in molecular pathways related to the immune system, kidney, and liver, which necessitate the need for personalized exposure monitoring (Gao et al., 2022).

Genetic information and exposure profiles are combined in customized risk frameworks. For instance, CYP1A1 and CYP1B1 enzyme activity may be altered in urban commuters who are frequently exposed to high amounts of PM2.5 and traffic-related nitrogen oxides, which may necessitate a reduction in tamoxifen dosing to maintain therapeutic efficacy and safety in those patients (Wang et al., 2022). Furthermore, CYP2E1 and CYP3A4 are jointly induced in chronic alcohol users who are also exposed to aflatoxin B1, increasing their risk of hepatotoxicity and necessitating improved screening procedures to identify liver damage early (Chen and Wei, 2015).

Therapeutic Optimization through exposure-Aware can be seen in several cases. For instance, Prescribing Proton pump inhibitor (PPI) selection accounts for CYP2C19 genotype and indole-3-carbinol intake from cruciferous vegetables, which induce competing metabolic pathways. Also, warfarin dose adjustments should take into account vitamin K dietary fluctuations with CYP4F2 status, thus stabilizing anticoagulation response (Gaikwad et al., 2018). Together, these examples demonstrate how taking into account exposure and genetic factors allows for a more reliable and effective prescription, which is a step toward truly holistic precision medicine.

## 7 Conclusion and future directions

Understanding the factors influencing drug metabolism is essential for optimizing therapeutic outcomes. This review underscores the central role of cytochrome P450 (CYP450) enzymes in drug biotransformation and reveals the profound yet often overlooked impact of the exposome—the cumulative lifetime environmental and endogenous exposures—on CYP450 activity.

The exposome dynamically modulates CYP450 expression and function through a complex interplay of external factors such as diet, pollutants, and lifestyle, as well as internal elements like the microbiome and pathological states. These diverse influences explain much of the interindividual variability in drug response beyond genetic predispositions alone. For example, dietary components like grapefruit and environmental toxins induce significant changes in CYP enzyme activity, while chronic conditions further alter metabolic capacity via inflammatory and oxidative pathways.

In addition to external environmental factors, the internal exposome—including biological elements such as the gut microbiome, inflammation, and disease states—plays a crucial role in modulating CYP450 enzyme activity and drug metabolism. These internal factors dynamically interact with external exposures and genetic predispositions, further contributing to the complexity and variability of drug response. Recognizing the importance of both internal and external exposomes is essential for a comprehensive understanding of personalized pharmacotherapy.

Looking forward, integrating exposomic data with genetic information heralds a transformative direction in personalized medicine. This holistic approach captures the multifaceted regulation of drug metabolism and holds promise for enhancing drug efficacy, reducing adverse reactions, and enabling truly individualized therapy.

Achieving this vision requires advanced multi-omics technologies paired with artificial intelligence to unravel the complex, time-dependent interactions within the exposome. These innovations will facilitate biomarker discovery, mechanistic insights, and real-time clinical application, moving pharmacology beyond a gene-centric paradigm.

In summary, the exposome’s influence on CYP450-mediated drug metabolism is intricate and pivotal. Embracing this complexity is key to advancing precision pharmacotherapy, ultimately leading to safer, more effective, and equitable healthcare tailored to each individual’s unique environmental and biological context.
